# Survival and genomic stability of *Yersinia enterocolitica* in environmental *Acanthamoeba* spp

**DOI:** 10.3389/fcimb.2025.1642352

**Published:** 2025-10-30

**Authors:** Paola Chiani, Valeria Michelacci, Elisabetta Delibato, Eleonora Ventola, Samra Mannan, Manuela Marra, Valentina Libri, Rubén L. Rodríguez-Expósito, Maria Reyes-Batlle, Isabel de Fuentes, Jacob Lorenzo-Morales, Stefano Morabito, Margherita Montalbano Di Filippo

**Affiliations:** ^1^ Department of Food Safety, Nutrition and Veterinary Public Health, Istituto Superiore di Sanità, Rome, Italy; ^2^ Core Facilities Technical-Scientific Service, Istituto Superiore di Sanità, Rome, Italy; ^3^ Instituto Universitario de Enfermedades Tropicales y Salud Pública de Canarias (IUETSPC), Universidad de La Laguna (ULL), San Cristóbal de La Laguna, Spain; ^4^ CIBER de Enfermedades Infecciosas (CIBERINFEC), Instituto de Salud Carlos III, Madrid, Spain; ^5^ Departamento de Obstetricia y Ginecología, Pediatría, Medicina Preventiva y Salud Pública, Toxicología, Medicina Legal y Forense y Parasitología, Universidad de La Laguna (ULL), San Cristóbal de La Laguna, Spain; ^6^ Servicio de Parasitología, Centro Nacional de Microbiología, Instituto de Ciencias de la Salud Carlos III, Madrid, Spain

**Keywords:** *Y. enterocolitica*, free living amoebae (FLA), food safety, zoonosis, *Y. enterocolitica* biotypes, *Acanthamoeba* spp.

## Abstract

**Introduction:**

Free-living amoebae (FLA) are widespread protozoa that can host bacterial pathogens, promoting their persistence in the environment. *Yersinia enterocolitica*, a foodborne zoonotic pathogen, has been detected within amoebae, but its intracellular dynamics remain unclear.

**Methods:**

In this study, we explored the interaction between three *Y. enterocolitica* strains—differing in biotype and virulence gene profile—and two *Acanthamoeba* spp.—a reference strain and a wild environmental isolate.

**Results and discussion:**

All strains were internalized and survived up to 8 days in the collection strain and 16 days in the wild isolate. Intracellular persistence did not affect amoebal integrity or bacterial virulence profiles. Whole genome sequencing (WGS) revealed high genomic stability across strains, though specific mutations—such as in the *igaA* gene, involved in stress response—emerged after persistence in the collection strain. These findings suggest that *Acanthamoeba* spp. not only shields *Y. enterocolitica* from environmental stress but may also influence its genome and adaptive potential. This work expands the current understanding of *Y. enterocolitica* biology and highlights the role of FLA as reservoirs and potential drivers of bacterial evolution. Their contribution to the bacteria persistence and gene exchange warrants further investigation, particularly in the context of antimicrobial resistance and food safety.

## Introduction

Free-living amoebae (FLA) are widespread protozoa found in a wide range of natural and human-made environments, where they prey on bacteria, fungi, and algae. Among pathogenic genera, *Acanthamoeba* and *Vermamoeba* are linked to ocular infections in contact lens users (Lorenzo-Morales et al., 2015; Moran et al., 2022), *Naegleria fowleri* causes primary amoebic meningoencephalitis (PAM) in immunocompetent individuals (Visvesvara et al., 2007), while *Balamuthia* and *Acanthamoeba* are responsible for granulomatous amoebic encephalitis (GAE) in immunocompromised patients (Kiderlen et al., 2014). FLA present two main life stages: the trophozoite, an active feeding form that consumes bacteria, viruses, algae, and yeast, and the cyst, the resistant structure that allows survival under adverse environmental conditions (i.e., nutrient scarcity, extreme temperatures, pH changes, gamma radiation, and water treatments). These amoebae not only are efficient bacterial predators but also act as crucial environmental reservoirs for pathogenic microorganisms. Some bacterial species evade predation by internalizing within amoebal trophozoites, forming endosymbiotic relationships that shield them from environmental stress (Henriquez et al., 2021; Dinda et al., 2024). Inside the vacuolar environment of the amoebae, these bacteria resist degradation, can replicate, and enhance their survival. This mechanism promotes both persistence and dissemination, raising the risk of human exposure. Additionally, amoebal endosymbiosis could drive horizontal gene transfer, contributing to antibiotic resistance and adaptation to hostile conditions (Greub and Raoult, 2004; Henriquez et al., 2021; Montalbano Di Filippo et al., 2022; Dinda et al., 2024).

Notably, several foodborne zoonotic agents, including *Salmonella enterica*, *Listeria monocytogenes*, and Shiga toxin-producing *Escherichia coli* (STEC), have been detected within amoebal hosts, where endosymbiosis enhances their resistance to disinfection and environmental stress (Vaerewijck et al., 2014; Chavatte et al., 2014; Scheid, 2018; Montalbano Di Filippo et al., 2022; Dinda et al., 2024). This relationship underscores the role of FLA as reservoirs that support the persistence and transmission of zoonotic pathogens in food processing and water distribution systems. Among foodborne pathogens, *Y. enterocolitica* represents a significant threat due to its zoonotic potential and ability to persist in various environments.

*Y. enterocolitica* is a foodborne zoonotic pathogen responsible for yersiniosis, the fourth most-reported foodborne zoonosis in Europe in 2023, with 8,738 cases (EFSA ECDC, 2024). It belongs to the *Yersiniaceae* family (28 species https://lpsn.dsmz.de/genus/yersinia—Adeolu et al., 2016), which also includes the pathogenic species *Y. pestis* and *Y. pseudotuberculosis* (Adeolu et al., 2016). The main route of human infection is through the consumption of contaminated food, particularly undercooked pork, but *Y. enterocolitica* has also been detected in milk, dairy products, fish, molluscs, vegetables, fruits, and water (Mancini et al., 2022; Mancusi et al., 2024). Although infections are typically self-limiting with gastrointestinal symptoms, severe complications like mesenteric lymphadenitis and terminal ileitis may occur, especially in children, the elderly, and immunocompromised individuals (Shoaib et al., 2019; Todd, 2014).

*Y. enterocolitica* displays considerable genetic and phenotypic diversity, classified into six biotypes (BT)—1A, 1B, 2, 3, 4, and 5—and approximately 70 serotypes (Fredriksson-Ahomaa, 2017). Pathogenicity varies significantly among biotypes: BT1A is generally non-pathogenic, BT1B is highly virulent, while BT2 to BT5 exhibit moderate pathogenic potential (Reuter et al., 2014). Pathogenic strains harbor the 70-kb virulence plasmid (pYV), which encodes essential virulence factors such as *yadA* (adhesin A) and *virF* (transcriptional regulator), alongside chromosomal genes *invA* (invasin), *ail* (attachment and invasion locus), *ystA* (*Yersinia* stable toxin A), and *myfA* (mucoid *Yersinia* factor A), contributing to its pathogenicity (Rivas et al., 2021). BT1A strains are generally considered non-pathogenic due to the absence of the pYV plasmid and key chromosomal virulence genes like *ail*. However, they may carry alternative virulence factors such as *ystB* (*Yersinia* stable toxin B) (Bhagat and Virdi, 2007).

Despite its relevance in food safety, interactions between *Y. enterocolitica* and FLA remain largely unexplored (Lambrecht et al., 2013; 2017). Understanding how *Y. enterocolitica* exploits FLA as environmental reservoirs could reveal mechanisms that enhance its resistance to disinfection and its persistence in adverse conditions. Filling this knowledge gap may lead to improved food safety protocols and reduced zoonotic transmission risks.

In this study, we applied a combined approach to explore the interaction between *Y. enterocolitica* and a reference and a wild strain of *Acanthamoeba* spp., used as a model organism. Specifically, we (i) evaluated the internalization capacity of *Y. enterocolitica* strains with distinct virulence gene profiles; (ii) determined whether the bacterium remains dormant or actively proliferates within the protozoan; and (iii) assessed the integrity and genomic stability of internalized *Y. enterocolitica* strains.

## Materials and methods

### Amoebal strains and culture conditions

For the invasion assays, two distinct *Acanthamoeba* strains were employed: (i) a reference strain (*Acanthamoeba* sp., CDC: V036, NR-46463) provided by BEI Resources, NIAID, NIH, and (ii) a wild-type isolate of *Acanthamoeba* spp. collected from a natural water source in Torrijos, Spain, kindly provided by our colleagues (co-authors of this work). The wild-type strain was cultured at 37°C and initially identified at the genus level by light microscopy. Molecular characterization was subsequently performed through conventional polymerase chain reaction (PCR) targeting the 18S rRNA gene, confirming its assignment as *Acanthamoeba* sp., genotype T4 (Schroeder et al., 2001).

Both strains were cultured axenically as monolayers in 25-cm^2^ tissue culture flasks using proteose peptone–yeast–glucose (PYG) medium, composed of 0.75% (w/v) proteose peptone, 0.75% (w/v) yeast extract, and 1.5% (w/v) glucose (Montalbano Di Filippo et al., 2022).

Cultures were maintained at 37°C under static conditions. Adherent amoebae were detached by vigorous tapping of the culture flasks, followed by centrifugation at 300 × *g* for 5 min. Cells were then washed with phosphate-buffered saline (PBS) and resuspended in fresh PYG medium. All experiments were performed with stationary-phase cultures (5–10 days old), containing approximately 95% trophozoites.

### Bacterial strains—culture conditions and genetic characterization

Three *Y. enterocolitica* strains were used in this study, representing both pathogenic and non-pathogenic biotypes. In particular, the reference strain *Y. enterocolitica* ATCC 23715 (bioserotype 1B/O:8) and strains Ye13P (bioserotype 4/O:3) and Ye15H (bioserotype 1A/O:5) were used. The latter two strains were isolated from human fecal samples of patients. The main characteristics of the strains, including bioserotype, source, country, year of isolation, and virulence markers, are summarized in **Table 1**.

**Table 1:** Characteristics of *Y. enterocolitica* strains used in this study.

*Yersinia* strains	Bioserotype	Source, country, year	Virulence profile
YeREF (ATCC 23715*)	1B/O:8	Human, United States, 1968	*ail*
Ye13P	4/O:3	Human, Italy, 2016	*ail; yadA* and *virF (pYV+)*
Ye15H	1A/O:5	Human, Italy, 2019	*ystB*

*Y. enterocolitica subsp. *enterocolitica* (Schleifstein and Coleman) Frederiksen (ATCC 23715).

The *Y. enterocolitica* strains were cultured in Tryptone Soy Broth (TSB, Biolife Italiana, Milan, Italy) and subsequently inoculated onto CIN Agar plates (Cefsulodin–Irgasan–Novobiocin, Biolife Italiana, Milan, Italy). Plates were incubated under aerobic conditions at 30°C for 24 ± 2 h. Biotyping was carried out according to the ISO 10273 method (EN ISO 10273:2017, 2017), evaluating specific biochemical reactions, including pyrazinamidase and lipase activity, indole production, xylose and trehalose fermentation, and esculin hydrolysis. For serotyping, O-antisera specific to O:3, O:5, O:8, O:9, and O:27 (Biolife Italiana, Milan, Italy) were employed.

The genetic characterization of the three *Y. enterocolitica* strains was performed using a SYBR Green-based real-time PCR, targeting the main virulence genes associated with pathogenicity. The amplification conditions and the melting curve analysis were conducted according to the protocols described by Ventola et al. (2023). Specifically, the presence of the *ail* gene was used as a marker for pathogenic strains, according to the ISO/TS 18867 method (ISO/TS 18867, 2015; Tast Lahti et al., 2023). The strain belonging to biotype 4/O:3 was further characterized by the detection of the virulence plasmid pYV, which encodes *virF* and *yadA*. In contrast, the non-pathogenic strain (1A/O:5) was identified by the exclusive presence of *ystB*, with no detection of *ail* or pYV.

### Antibiotic susceptibility of *Yersinia enterocolitica*

To optimize the conditions for the invasion assay, different antibiotics were tested to determine their ability to inhibit *Y. enterocolitica* growth. Pathogenic and non-pathogenic *Y. enterocolitica* strains were cultured in TSB until reaching an optical density (OD) of 0.5, then distributed into 96-well plates and exposed to different concentrations of tetracycline, gentamicin, penicillin/streptomycin, and kanamycin.

The antibiotic treatments were evaluated at different time points: 1 h, 2 h, 3 h, 4 h, and overnight. Following each interval, the cultures were plated onto CIN Agar to assess bacterial survival.

### Intracellular survival assays

The ability of *Y. enterocolitica* to survive and persist within *Acanthamoeba* spp. was evaluated using intracellular survival assays performed two technical replicates per condition within a single representative experiment, following the protocol described by Montalbano Di Filippo et al. (2022). *Acanthamoeba* spp. were seeded into 48-well plates containing PYG medium and incubated at 37°C for 24–48 h to reach at least 70% confluency. After incubation, the medium was removed, and the wells were gently washed with PBS. *Y. enterocolitica* strains were then added [10^6^ colony-forming units (CFU)/mL is equal to a multiplicity of infection (MOI) of 1], and multi-well plates were incubated at 30°C (optimal *Y. enterocolitica* growth temperature).

The infection assay was monitored for a total of 55 days, with samples collected at defined time points: 3, 8, 16, 21, and 55 days post-infection. At each time point, the culture medium was carefully inspected, and antibiotic (see details in the Results section) was added to eliminate extracellular bacteria, followed by overnight incubation at 30°C.

To recover intracellular bacteria, *Acanthamoeba* spp. were washed rigorously three times with PBS to remove any residual of extracellular bacteria and antibiotics and lysed with sodium dodecyl sulfate (SDS; 0.5% final concentration) for 40 min. A portion of the resulting lysate was plated onto CIN Agar and incubated at 30°C for 24 h to quantify CFU, while the remaining lysate was stored at −20°C for subsequent molecular analysis. If *Y. enterocolitica* colonies were observed on CIN plates, a randomly selected colony was used as template for real-time PCR to confirm the presence of virulence genes, following the protocol described in the Bacterial strains—culture conditions and genetic characterization section. The assays were used for qualitative confirmation purposes only and were not intended to measure gene expression levels.

When no colonies were observed on CIN Agar, the stored lysate was subjected to real-time PCR targeting strain-specific virulence genes to inspect the presence of *Y. enterocolitica* DNA inside *Acanthamoeba* spp. (data not shown). This additional analysis aimed to assess whether bacterial DNA persisted within the amoebal lysate, suggesting that the absence of growth might be due to loss of viability during SDS lysis rather than lack of internalization.

As a rule, all experiments included *Y. enterocolitica* cultures grown in the absence of *Acanthamoeba* spp. as negative controls, subjected to the same experimental conditions.

### Whole genome sequencing of the internalized *Yersinia enterocolitica*

In order to assess genome integrity and stability, 14 *Y. enterocolitica* strains were subjected to whole genome sequencing (WGS), including wild-type strains YeREF, Ye13P, and Ye15H strains grown in broth for 24 h, 8 days, and 16 days and five strains that had successfully internalized into both *Acanthamoeba* spp. strains and were recovered from agar plates. Total DNA was extracted from 2 mL of overnight culture in TSB at 37°C using the GRS Genomic DNA Kit for Bacteria (GRISP Research Solutions, Porto, Portugal). Sequencing was performed using Ion Torrent S5 and S5 Prime platforms (Thermo Fisher Scientific, MA, USA). Libraries of approximately 400 bp were prepared from 100 ng of genomic DNA using the NEBNext^®^ Fast DNA Fragmentation & Library Prep Set for Ion Torrent™ (#E6285L, New England BioLabs, MA, USA). Library quality and quantity were evaluated with the Agilent TapeStation 4200 system using the High Sensitivity D1000 Reagents Kit (#5067-5585, Agilent Technologies). Enrichment was carried out on the Ion Chef System with the Ion 510™, 520™, and 530™ Kits (#A34018, #A27754, and #A27755, Thermo Fisher Scientific), followed by sequencing.

### Genomic characterization of the *Yersinia enterocolitica* strains used in the internalization experiments

The bioinformatic analyses were carried out using the tools present in the Galaxy public server ARIES (https://w3.iss.it/site/aries/) (Knijn et al., 2020). The reads were assembled in contigs using SPAdes v3.14.1 (Bankevich et al., 2012), and the contigs were filtered with the tool “Filter SPAdes repeats” (Iskander, 2022) by using default parameters. The virulence gene content of the genomes was determined using blastn through ARIES with an in-house developed sequence database (accession numbers: *ail*, CP009846; *virF*, AF336309; *yadA*, CP009845; and *ystB*, D88145), by using 85% of sequence identity and 70% alignment length as thresholds. Phylogenomics analysis was performed by determining the core genome multilocus sequence typing (cgMLST) using the chewBBACA tool (Silva et al., 2018) and the scheme developed by the INNUENDO project, which comprises 2,406 loci in total (Llarena et al., 2018; Mirko et al., 2018). The distances between strains were calculated by pairwise comparison of the allelic profiles through the chewTree tool (https://www.iss.it/site/aries). The pairwise comparison was considered reliable when >90% of loci for each sample were assigned to an allele. For each pair of samples, the alleles that were not found, only partially found, or not correctly assigned to any locus were excluded from the analysis, as previously described (Gigliucci et al., 2021). Genome annotation was performed with the Prokka tool (Seemann, 2014) on the ARIES server.

## Results

### *Yersinia enterocolitica* survival within *Acanthamoeba* spp.

Before presenting our results, it is important to note that among the antibiotics tested, only gentamicin fully suppressed bacterial growth after overnight incubation (optimal concentration of 350 µg/mL).

Our findings showed that all tested *Y. enterocolitica* strains were internalized by both *Acanthamoeba* species and remained viable for different timeframes, depending on the strain and the amoebal host. Viability was maintained for up to 8 days in *Acanthamoeba* spp. CDC: V036 and for up to 16 days in the wild-type isolate. Further details are provided in **Table 2**. Microscopic examination confirmed that *Acanthamoeba* spp. strains remained structurally intact throughout the entire incubation period at 30°C, even in the absence of nutrient supplementation. Furthermore, all internalized *Y. enterocolitica* strains retained their original virulence gene profiles, as confirmed by real-time PCR (data not shown), indicating preservation of their pathogenic potential. In detail, all the tested recovered colonies of the Ye13P strain were positive for *ail*, *yadA*, and *virF* genes; all those of the YeREF strain were positive for the *ail* gene; and all those of the Ye15H strain were positive for the *ystB* gene only.

**Table 2:** Intracellular survival of *Y. enterocolitica* within *Acanthamoeba* spp.

*Ye*REF										
Time points	3 days		8 days		16 days		21 days		55 days	
Replicates	R1	R2	R1	R2	R1	R2	R1	R2	R1	R2
*Acanthamoeba* collection strain (CDC: V036)	143 CFU	165 CFU	1 CFU	1 CFU	0 CFU	0 CFU	0 CFU	0 CFU	0 CFU	0 CFU
*Acanthamoeba* sp. (wild isolate)	105 CFU	175 CFU	57 CFU	220 CFU	4 CFU	0 CFU	0 CFU	0 CFU	0 CFU	0 CFU

*Ye*13P										
Time points	3 days		8 days		16 days		21 days		55 days	
Replicates	R1	R2	R1	R2	R1	R2	R1	R2	R1	R2
*Acanthamoeba* collection strain (CDC: V036)	>1,000 CFU	>1,000 CFU	240 CFU	110 CFU	0 CFU	0 CFU	0 CFU	0 CFU	0 CFU	0 CFU
*Acanthamoeba* sp. (wild isolate)	0 CFU	24 CFU	57 CFU	220 CFU	0 CFU	0 CFU	1 CFU*	0 CFU	0 CFU	0 CFU

*Ye*15H										
Time points	3 days		8 days		16 days		21 days		55 days	
Replicates	R1	R2	R1	R2	R1	R2	R1	R2	R1	R2
*Acanthamoeba* collection strain (CDC: V036)	170 CFU	>1,000 CFU	1 CFU	1 CFU	0 CFU	0 CFU	0 CFU	0 CFU	0 CFU	0 CFU
*Acanthamoeba* sp. (wild isolate)	>1,000 CFU	>1,000 CFU	2 CFU	9 CFU	0 CFU	3 CFU	0 CFU	0 CFU	0 CFU	0 CFU

CFU reported are in duplicate from a single representative experiment. The asterisk (*) highlighted bacterial growth unrelated to *Y. enterocolitica* but identified through sequencing as *Acinetobacter ursingii* (data not shown).

### Genomic characterization of the internalized *Yersinia enterocolitica* strains

The median number of contigs obtained from the genome assemblies was 64, with a median N50 value of 140,608 bp, indicating high-quality assemblies across all samples. The expected virulence genes could be retrieved in the genomes for all the strains analyzed. In detail, blast analysis confirmed that the YeREF strain was positive for the *ail* gene either after growth in broth for 24 h, 8 days, or 16 days or when recovered after internalization in the *Acanthamoeba* collection strain for 8 days and in the wild *Acanthamoeba* strain for 16 days; similarly, the Ye13P strain was positive for *ail*, *yadA*, and virF either after growth in broth for 24 h, 8 days, or 16 days or when recovered after internalization in the *Acanthamoeba* collection strain for 8 days; finally, Ye15H was positive for *ystB* either after growth in broth for 24 h, 8 days, or 16 days or when recovered after internalization in the *Acanthamoeba* collection strain for 8 days and in the wild *Acanthamoeba* strain for 16 days.

To evaluate genome integrity and stability following intracellular persistence, a comparative phylogenetic analysis was performed using cgMLST. A minimum of 2,348 allelic exact matches were detected in all the genomes tested among the 2,406 loci part of the scheme used.

**Figure 1:**
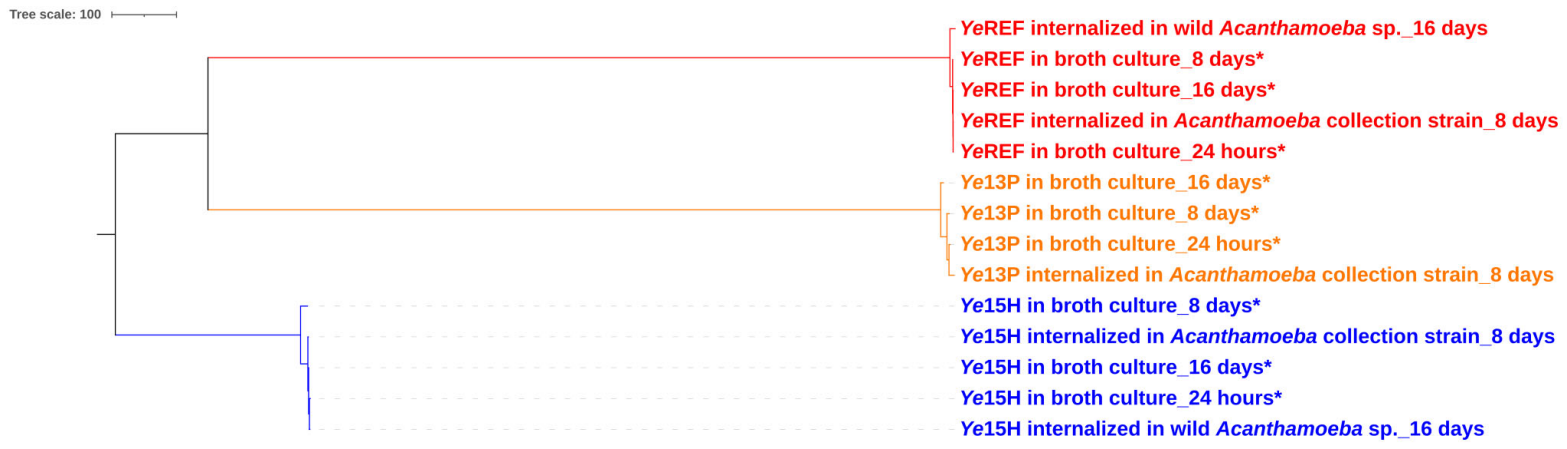
Phylogenomic analysis of *Y. enterocolitica* strains analyzed in this study—both those recovered under standard laboratory conditions (broth culture—highlighted by a star mark) and those following internalization within amoebal hosts, through cgMLST. Clade colors denote bacterial pathogenicity (red: pathogenic, orange: moderately pathogenic, and blue: non-pathogenic); pathogenicity was determined based on the detection of known virulence genes and serotype information as reported in the Materials and Methods section.

For comparison, the same *Y. enterocolitica* strains cultured in broth under standard laboratory conditions for 16 days were included in the analysis. The resulting allelic distance-based dendrogram was visualized and modified using iTOL (https://itol.embl.de/; Letunic and Bork, 2007) and is shown in **Figure 1**.

The analysis revealed three distinct clades corresponding to the strains under study: YeREF (*Y. enterocolitica* ATCC 23715 1B/O:8), Ye13P (*Y. enterocolitica* 4/O:3), and Ye15H (*Y. enterocolitica* 1A/O:5). Each clade exhibited minimal variability, suggesting a relatively high level of genomic stability during intracellular persistence within *Acanthamoeba* spp. Specifically, the maximum number of allelic differences (ADs) detected among isolates internalized for 8 days in *Acanthamoeba* spp. CDC: V036 was 10, detected in Ye13P.

Notably, no allelic variation was detected in strains recovered from wild *Acanthamoeba* spp., whereas isolates internalized within the reference strain (*Acanthamoeba* spp. CDC: V036) accumulated a moderate number of mutations, exceeding those observed under standard laboratory (broth) conditions. In detail, (i) Ye13P accumulated 6 ADs after 8 days in broth and 10 ADs after 8 days within the reference strain of amoeba. Six loci were shared between both conditions, while four additional loci were uniquely mutated during the intracellular persistence: in detail, *dusB* (tRNA-dihydrouridine synthase B), *ravA* (ATPase RavA), *igaA* (intracellular growth attenuator protein IgaA), and *rnb* (Exoribonuclease 2); (ii) Ye15H showed no ADs after 8 days in broth, but displayed 2 ADs following internalization in the CDC: V036 host, specifically in *manA* (Mannose-6-phosphate isomerase) and *tap* (Methyl-accepting chemotaxis protein IV).

Interestingly, a mutation in the *igaA* gene was identified in strain Ye13P exclusively following interaction with the *Acanthamoeba* CDC: V036 host. This gene encodes an intracellular growth attenuator described in *S. enterica*, where it is involved in the negative regulation of the Rcs phosphorelay system, a key pathway controlling bacterial stress responses and virulence (Dominguez-Bernal et al., 2004). However, the *igaA* locus was not detected at all in the Ye13P genome after growing in broth for 8 days.

## Discussion

FLA often act as environmental shelters that enhance the survival of foodborne zoonotic pathogens under adverse conditions. *Y. enterocolitica* has been shown to benefit from this interaction, with early work by King et al. (1988) demonstrating increased chlorine resistance following internalization by *Acanthamoeba castellanii*. Later studies proposed a possible mutualistic relationship under nutrient-rich conditions, allowing bacterial survival without damaging the host (Greub and Raoult, 2004; Siddiqui and Khan, 2012).

Experimental studies confirmed that *Y. enterocolitica* can persist in amoebal hosts under multiple stress conditions, including long-term intracellular survival (up to 14 days) and viability at temperatures simulating refrigeration (Anacarso et al., 2011; Lambrecht et al., 2013). Internalization has also been associated with increased resistance to disinfectants like chlorine, raising concerns for sanitation protocols also in industrial settings (Thomas et al., 2010; Vaerewijck et al., 2014; Lambrecht et al., 2017).

Together, these findings highlight the ecological and public health relevance of *Y. enterocolitica* and FLA interactions. However, important gaps remain regarding the effect of internalization on strains with different virulence profiles, their intracellular fate, and the consequences for structural and genetic stability. To address these aspects, we explored the intracellular dynamics of different *Y. enterocolitica* isolates using two *Acanthamoeba* spp. strains: one collection-derived and one field-isolated.

Our results confirmed the ability of all tested *Y. enterocolitica* strains to remain viable after internalization, with intracellular persistence observed up to 8 days in the collection-derived *Acanthamoeba* spp. strain and extending to 16 days in the wild-type isolate; this extends the findings of Lambrecht et al. (2013), who reported viability within *A. castellanii* for up to 14 days, and suggests that host origin may influence the duration of bacterial survival. Our morphological analysis revealed that both amoebal strains remained structurally intact at 30°C throughout the incubation period, even in the absence of nutrient supplementation, corroborating earlier reports on the stability and resilience of *Acanthamoeba* spp. as a protozoan host (Greub and Raoult, 2004; Siddiqui and Khan, 2012).

Importantly, real-time PCR and genome analysis demonstrated the conservation of virulence gene profiles in all internalized *Y. enterocolitica* strains. This observation supports the idea that intracellular persistence does not compromise the pathogenic potential of the *Y. enterocolitica*, in contrast with scenarios in which intracellular niches lead to attenuation or gene loss. While this aspect had not been extensively addressed in previous studies on *Y. enterocolitica*, similar findings have been reported in *S. enterica* and in STEC, where intracellular maintenance in protozoa did not alter key virulence traits (Gaze et al., 2003; Montalbano Di Filippo et al., 2022).

However, WGS revealed that internalization may induce subtle genetic changes depending on the amoebal host. Indeed, no allelic variation was observed in bacteria recovered from the wild-type *Acanthamoeba* spp. strain, while ADs were detected in isolates internalized within the culture-derived strain, suggesting that wild amoebal strains could serve as reservoirs where genetic stability of *Y. enterocolitica* strains is preserved. The detected differences included a mutation in the *igaA* gene of strain Ye13P, detected exclusively after interaction with *Acanthamoeba* CDC: V036. This gene has been described in *S. enterica* to encode an inner membrane protein involved in the negative regulation of the Rcs phosphorelay system, a key regulator of envelope stress responses and virulence factor expression in enterobacteria (Dominguez-Bernal et al., 2004). This system has been implicated in bacterial adaptation to intracellular environments and biofilm formation (Clarke, 2010; Wall et al., 2020). Notably, the *igaA* locus became undetectable in Ye13P cultured in broth under standard laboratory conditions for the same time of interaction with the amoebal strain, suggesting that this locus could be particularly unstable and subjected to growing conditions. Further analysis would be necessary to investigate the role of this gene in the intracellular survival of *Y. enterocolitica* and in virulence regulation.

Taken together, our findings, in line with previous literature (Montalbano Di Filippo et al., 2022), support the hypothesis that *Acanthamoeba* spp. does not merely provide physical protection for bacterial pathogens, including *Y. enterocolitica*, but may also act as a selective microenvironment capable of modulating bacterial genome stability. Moreover, survival in *Acanthamoeba* species could allow *Y. enterocolitica* to escape detection in animal samples or in the vehicles of infection, while maintaining intact its virulence gene asset, as observed for the Ye13P pathogenic strain. While these results contribute to a broader understanding of amoebae as environmental reservoirs, they primarily expand current knowledge on *Y. enterocolitica* biology, particularly in relation to its genomic and functional plasticity in protozoan hosts.

In this context, amoebae should be increasingly considered as dynamic hosts with the potential to influence bacterial evolution. Their capacity to internalize diverse microbial taxa, maintain structural integrity under stress, and potentially mediate gene exchange through mechanisms such as outer membrane vesicles or microvesicles warrants further investigation.

## Data Availability

The datasets presented in this study can be found in online repositories. The names of the repository/repositories and accession number(s) can be found below: https://www.ncbi.nlm.nih.gov/, PRJEB89775.
